# Retrospective multicenter registry for endovascular treatment with newer devices in over 25‐cm femoropopliteal artery disease: A retrospective observational study

**DOI:** 10.1002/hsr2.1003

**Published:** 2022-12-20

**Authors:** Kazunori Horie, Mitsuyoshi Takahara, Tatsuya Nakama, Akiko Tanaka, Kazuki Tobita, Naoki Hayakawa, Shinsuke Mori, Yo Iwata, Kenji Suzuki

**Affiliations:** ^1^ Department of Cardiovascular Medicine Sendai Kousei Hospital Miyagi Japan; ^2^ Department of Metabolic Medicine Osaka University Graduate School of Medicine Osaka Japan; ^3^ Department of Cardiology Tokyobay UrayasuIchikawa Medical Center Chiba Japan; ^4^ Department of Cardiology Shonan Kamakura General Hospital Kanagawa Japan; ^5^ Department of Cardiovascular Medicine Asahi General Hospital Chiba Japan; ^6^ Department of Cardiology Saiseikai Yokohama City Eastern Hospital Kanagawa Japan; ^7^ Department of Cardiology Funabashi Municipal Medical Center Chiba Japan; ^8^ Department of Cardiology Tokyo Saiseikai Central Hospital Tokyo Japan

**Keywords:** angioplasty, drug eluting stents, endovascular grafting, infrainguinal endovascular, stenting, vascular centers

## Abstract

**Background and Aims:**

Endovascular therapy (EVT) is recommended in femoropopliteal (FP) lesions shorter than 25 cm by current guidelines; however, diffuse FP lesions remains challenging for EVT. The aim of this study was to investigate the efficacy of EVT with the latest devices for FP lesions longer than 25 cm.

**Methods:**

This retrospective multicenter registry analyzed patients presented peripheral artery disease (PAD) having FP lesions longer than 25 cm who underwent EVT using the latest devices between 2017 and 2021. The primary outcome was restenosis 1 year after EVT.

**Results:**

The present study enrolled a total of 504 PAD patients with 614 lesions undergoing EVT for diffuse FP lesions. The Kaplan–Meier analysis showed that the rates of freedom from restenosis and clinically‐driven target lesion revascularization were 79.3% and 82.4% 1 year after EVT, respectively. The multivariate Cox proportional hazards regression analysis showed that clinical features associated independently with restenosis risk were cilostazol use (adjusted hazard ratio, 0.49 [0.32–0.74]; *p* = 0.001), reference vessel diameter (RVD) (0.72 [0.58–0.89] per 1‐mm increase; *p* = 0.002), and P3 segment involvement (2.08 [1.33–3.26]; *p* = 0.001). The Kaplan–Meier analysis was conducted to compare the primary patency between cases with and without a small RVD, P3 involvement, and/or lack of cilostazol; any risk factors were related to a worse primary patency rate, compared with cases without risk factors.

**Conclusion:**

In the current EVT era, the primary patency at 1 year was acceptable at 79.3% in patients with FP lesions longer than 25 cm. A small vessel and P3 segment involvement might be associated with a poor 1‐year patency rate after EVT, whereas cilostazol administration might contribute to reducing restenosis.

## 1 INTRODUCTION

Recently, the evolution of endovascular treatment (EVT) devices aimed at reducing reintervention rates in femoropopliteal (FP) lesions,^1^, ^2^, ^3^ and the European Society of Cardiology guidelines published in 2017 recommended EVT for FP lesions shorter than 25 cm as class I indication.^4^ However, the prevalence of peripheral artery disease (PAD) is largely increasing worldwide because of aging patient population and diabetes pandemic.^5^, ^6^ Patients with symptomatic PAD have had more complex FP lesions such as chronic total occlusion (CTO), severe calcification, and diffuse lesions.

The guideline also recommends bypass surgery (BSX) using a saphenous vein graft as the first‐choice treatment in patients with complex FP lesions, whereas suggested EVT for patients unsuitable for BSX with diffuse FP lesions, especially those longer than 25 cm (class IIb indication).^4^ However, aging and presence of multiple comorbidities make BSX difficult in such patients. Therefore, in daily practice, EVT is often chosen to treat patients with both complex FP lesions and several comorbidities. The aim of this study was to evaluate the safety and efficacy of the contemporary EVT using the latest devices in patients with symptomatic PAD having FP lesions longer than 25 cm in clinical practice. In addition, several studies indicated that medical treatment might be helpful to keep patency after revascularization such as antiplatelet therapy, cilostazol and oral anti‐coagulation; therefore, anti‐thrombotic therapies can play an important role in the current EVT era.^7^, ^8^, ^9^ We investigated the associated factors to improve the vessel patency, which included patent and lesion background, EVT devices and medical treatment.

## 2 MATERIALS AND METHODS

### 2.1 Study design, participants, and inclusion/exclusion criteria

**Figure 1:**
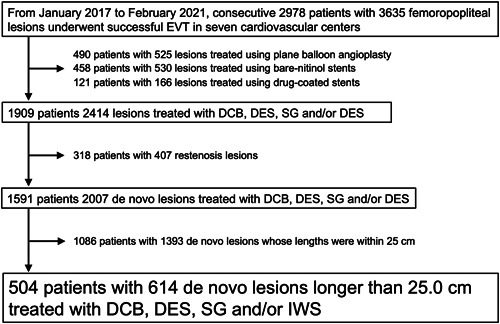
Study flow chart. DCB, drug‐coated balloon; DES, drug‐eluting stent; EVT, endovascular therapy; IWS, interwoven nitinol stent; SG, stent‐graft.

Peripheral arterY intervention THerapy in lONg femoropopliteal lesions (PYTHON) study is a retrospective multicenter study enrolling patients with Rutherford class 2 to 6 PAD having FP lesions longer than 25 cm who underwent EVT with either drug‐eluting stents (DES), interwoven nitinol stents (IWS), drug‐coated balloons (DCB) or intraluminal stent‐graft (SG) between December 2017 and February 2021. Patients with (1) nonatherosclerotic disease, (2) life expectancy <12 months, (3) acute limb ischemia, (4) in‐stent restenosis, and (5) aneurysmal lesions were excluded. From the database of seven cardiovascular centers including consecutive 3635 patients with FP lesions, a total of 504 patients with 614 lesions undergoing EVT for diffuse FP lesions at each hospital were retrospectively reviewed (Figure 1).^10^

The local ethics committees of all participating centers approved the protocol of the present study. This study was conducted according to the Declaration of Helsinki. As this study was retrospective, the written informed consent was not obtained, and the clinical data was collected from existing medical records. Alternatively, patients could opt out of the study. Relevant information regarding the study can be available to the public in accordance with Health the Research Involving Human Subjects and Ethical Guidelines for Medical.

### 2.2 Procedures

Following local anesthesia, a 5.0‐ to 7.0‐French guiding sheath was inserted into a common femoral artery. In CTO lesions, the wire‐crossing technique was dependent on the discretion of each operator. The bidirectional approach was performed as needed. The types of finalized devices were also dependent on decision of operators at each institution. Since atherectomy and intravascular lithotripsy devices were not commercially available in our country during the study period, these devices were not used.

### 2.3 Antithrombotic treatment

Dual antiplatelet therapy (DAPT; aspirin with clopidogrel or prasugrel) was prescribed for at least 1 week before the EVT and continued at least 1 month.^11^ When patients had bleeding complications or high bleeding risks, single antiplatelet therapy (SAPT) could be allowed.^4^ Subsequent prescription of the antiplatelet therapy was at the discretion of each treating physician. Oral anticoagulation was prescribed in patients with deep vein thrombosis, atrial fibrillation or heart valve implantation. In patients with oral anticoagulation, additional SAPT was recommended before and after the EVT.^4^

### 2.4 Study definition

This study included patients with FP lesions longer than 25 cm. Procedural success was defined as residual stenosis less than 50%. The primary outcome was primary patency within 1 year, defined as the absence of both restenosis and revascularization of the treated lesion. Restenosis was defined as a peak systolic velocity ratio of over 2.4 measured using duplex ultrasound, >50% diameter stenosis or occlusion by follow‐up angiography.^12^ The secondary outcomes included overall survival (freedom from all‐cause mortality), limb salvage (freedom from major amputation), and freedom from clinically‐driven target lesion revascularization (CD‐TLR). Color Doppler ultrasonography was routinely performed at 6 and 12 months after EVT to assess arterial patency, as recommended by the reports from the Society for Vascular Surgery.^13^ CD‐TLR referred to any revascularization procedures for significant restenosis and/or reocclusion within 1 year after EVT, as identified by duplex ultrasonography or angiography, with recurrent symptoms. The Peripheral Arterial Calcium Scoring System was used to categorize the severity of lesion calcification.^14^

### 2.5 Data analysis and statistical methods

The present study was analyzed according to the Statistical Analyses and Methods in Published Literature guideline and the guidance of the proper clinical research.^15^, ^16^ Baseline characteristics are presented as the mean ± standard deviation for continuous variables and the frequency (percentage) for categorical variables, if not otherwise mentioned. A two‐sided *p* of less than 0.05 was considered statistically significant, and 95% confidence intervals (CIs) are reported when appropriate. The rates of freedom from events were estimated using the Kaplan–Meier analysis. Baseline characteristics that were associated with the 1‐year restenosis risk was explored using the multivariate Cox proportional hazards regressions analysis. The variables showing significance in the univariate Cox proportional hazards regression model were entered in the multivariate model. We supplementally demonstrated the Kaplan−Meier analysis for freedom from restenosis of the subgroups into which the study population was divided according to both modifiable and nonmodifiable risk factors for restenosis that were identified in the multivariate regression model. All statistical analyses were performed with R version 4.1.1 (R Development Core Team).

## 3 RESULTS

**Figure 2:**
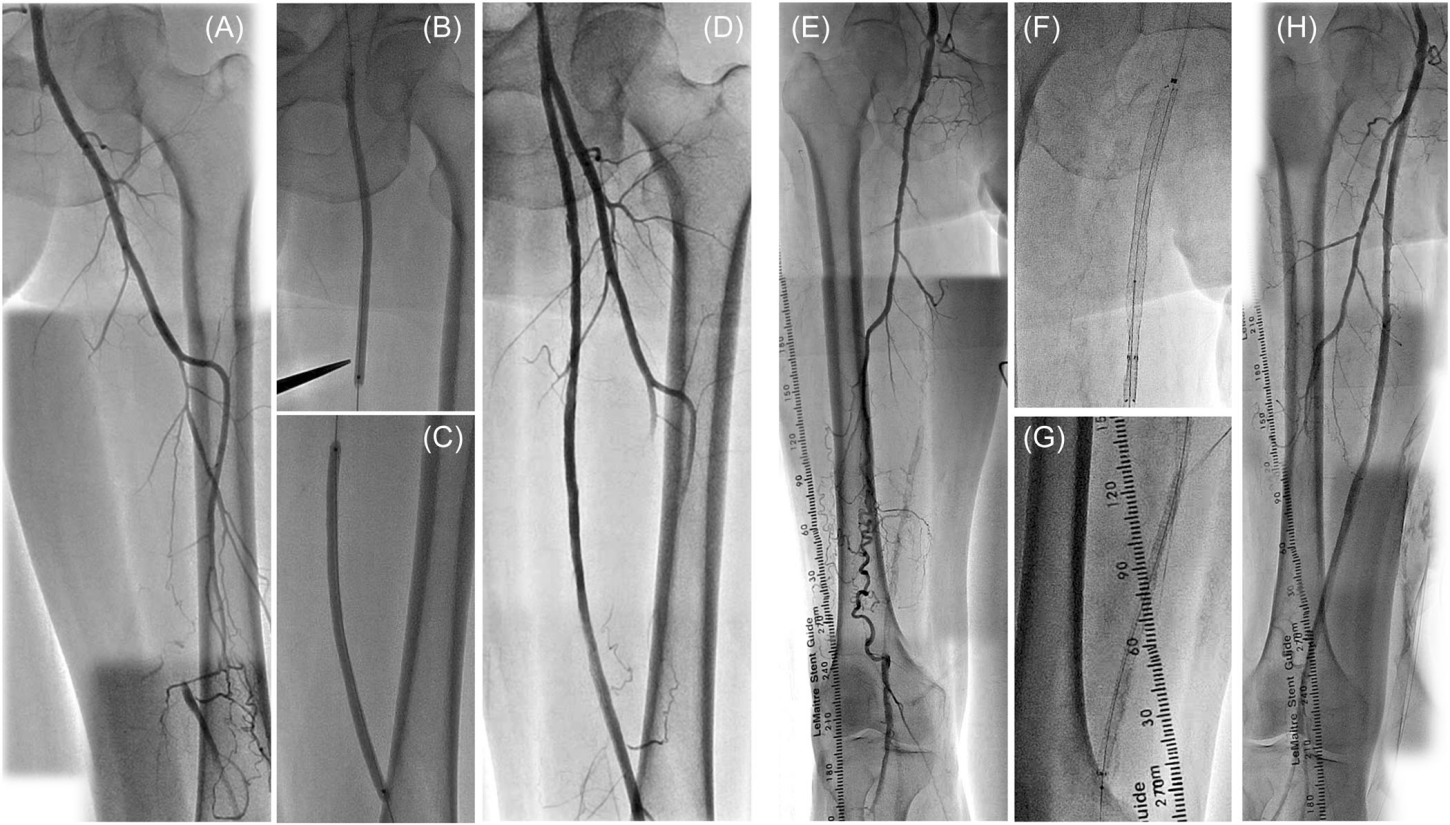
Representative cases with diffuse femoropopliteal lesions treated with endovascular therapy using current devices. (A) A 73‐year‐old male with uncontrolled diabetes complained of Rutherford class III claudication. Baseline angiography showed 27 cm chronic total occlusion (CTO) in his left superficial femoral artery (SFA). (B–C) Two 5.0 × 150 mm drug‐coated balloons were dilated in the CTO. (D) Final angiography revealed that residual stenosis was less than 50% after dilatation. (E) A 78‐year‐old male with end‐stage renal disease receiving hemodialysis complained of intractable rest pain. Baseline angiography showed 32 cm CTO with severe calcification from his right SFA to proximal popliteal artery. (F−G) A 6.0 × 250 mm covered stent graft and a 7.0 × 120 mm drug‐eluting stent were implanted in the femoropopliteal segment. (H) Final angiography revealed sufficient antegrade blood flow without significant residual stenosis.

The clinical characteristics are summarized in Table 1. The mean patient age was 76 ± 9 years, and the rate of male patients were 62.9% (317/504). The rates of diabetes mellitus and end‐stage renal disease requiring dialysis were 62.7% and 27.4%, respectively. Approximately one‐third of the patients had chronic limb‐threatening ischemia (35.7%). The mean reference vessel diameter (RVD) and lesion length were 5.4 ± 0.8 mm and 30.6 ± 5.1 cm, respectively. The CTO and bilateral wall calcification were observed in 70.0% and 48.5% of the study lesions, respectively. Combined use of finalized devices was performed in 19.9%. Figure 2 shows representative cases with FP lesions longer than 25 cm undergoing successful EVT using DCB (Figure 2A−D) or combination of intraluminal SG and DES (Figure 2E−H).

**Table 1:** Baseline characteristics of the study population

Patient characteristics	(*n* = 504)
Male sex	317 (62.9%)
Age (years)	76 ± 9
Diabetes mellitus	316 (62.7%)
Smoking	115 (22.8%)
Renal failure on dialysis	138 (27.4%)
Aspirin use	413 (81.9%)
P2Y12 antagonist use	442 (87.7%)
Dual antiplatelet therapy use including aspirin and P2Y12 antagonist	363 (72.0%)
Cilostazol use	169 (33.5%)
Anticoagulant use	89 (17.7%)
Limb characteristics	(*n* = 614)
Chronic limb‐threatening ischemia	219 (35.7%)
Ankle brachial index	0.54 ± 0.26
(missing data)	37 (6.0%)
History of endovascular therapy	60 (9.8%)
Popliteal involvement	349 (56.8%)
P3 involvement	97 (15.8%)
Reference vessel diameter (mm)	5.4 ± 0.8
Lesion length (mm)	305.8 ± 51.2
Chronic total occlusion	430 (70.0%)
PACSS classification	
Grade 0	184 (30.0%)
Grade 1	86 (14.0%)
Grade 2	46 (7.5%)
Grade 3	50 (8.1%)
Grade 4	248 (40.4%)
Below‐the‐knee runoff	
No runoff	40 (6.5%)
1 runoff	277 (45.1%)
2 runoffs	213 (34.7%)
3 runoffs	84 (13.7%)
DCB use	258 (42.0%)
DES use	257 (41.9%)
Stent graft use	153 (24.9%)
IWS use	76 (12.4%)
Combined use of finalized devices	122 (19.9%)
Intravascular utlrasound use	500 (81.4%)

*Note*: Categorical variables are expressed as number and percentage. Continuous variables are indicated as mean ± standard deviation or median (interquartile range).

Abbreviations: DCB, drug‐coated balloon; DES, drug‐eluting stent; IWS, interwoven nitinol stent; PACSS, Peripheral Arterial Calcium Scoring System.

**Figure 3:**
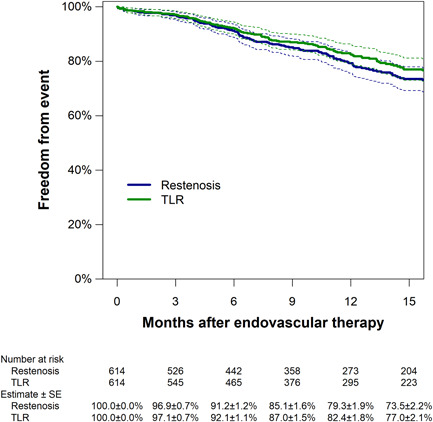
Kaplan−Meier estimates of freedom from restenosis and target lesion revascularization (TLR). Dotted lines indicate 95% confidence intervals.

**Figure 4:**
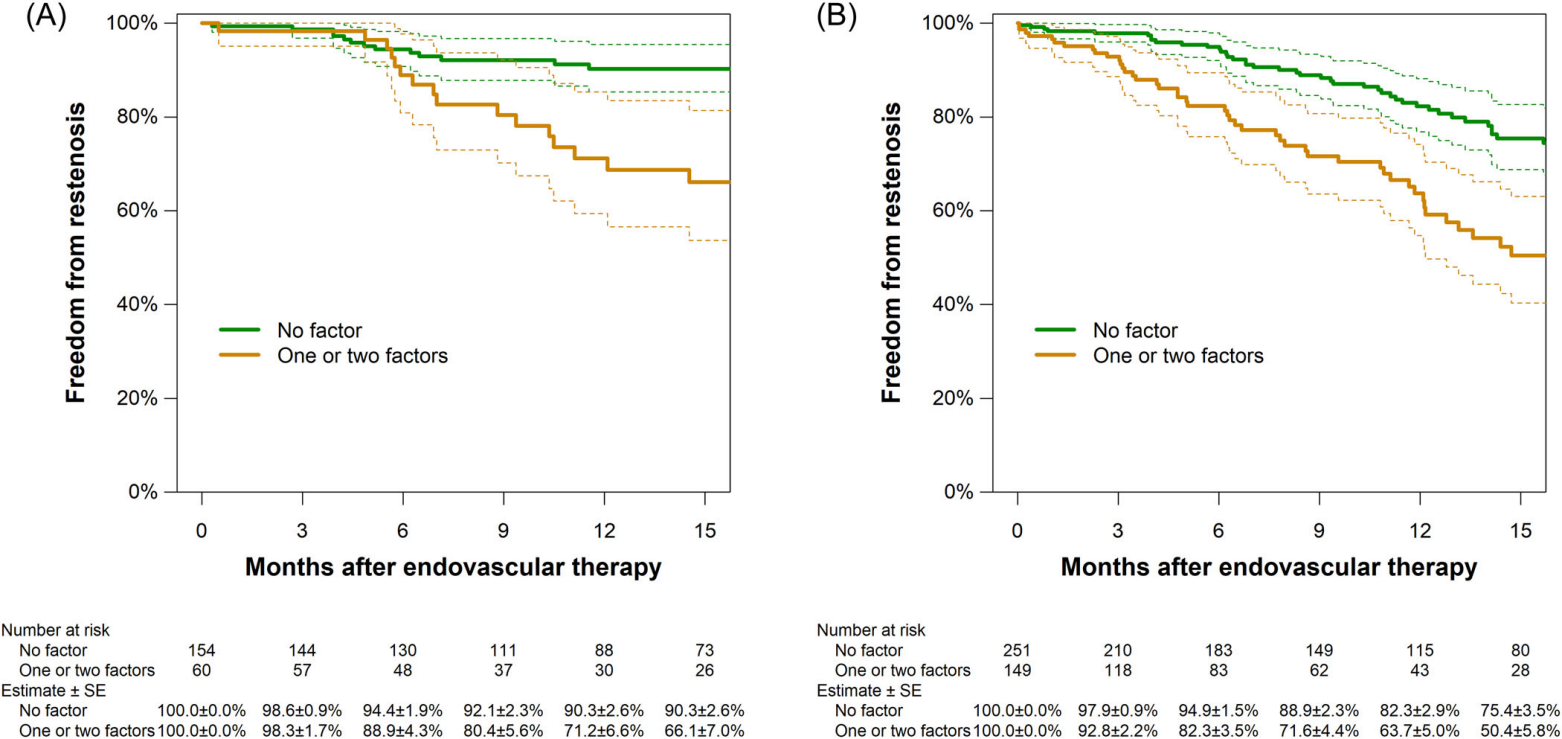
Kaplan−Meier estimates of freedom from restenosis by lesion risk factors in a population with (A) and without cilostazol use (B). Lesion risk factors for restenosis were P3 involvement and reference vessel diameter < 5 mm (see Table 2). Dotted lines represent 95% confidence intervals.

During a median follow‐up period of 10.7 (5.2–17.8) months (interquartile range), restenosis was detected in 135 cases. Figure 3 shows the Kaplan–Meier analysis of freedom from restenosis and CD‐TLR. The 1‐year rate of freedom from restenosis (i.e., primary patency) was 79.3% (95% CI, 75.6%–83.1%) and that of freedom from CD‐TLR was 82.4% (79.0%–85.9%). The association of clinical baseline characteristics and treatment findings with restenosis risk are shown in Table 2. The multivariate Cox proportional hazards regression analysis shows that clinical features independently associated with restenosis were cilostazol use (adjusted hazard ratio, 0.49 [0.32–0.74]; *p* = 0.001), P3 segment involvement (2.08 [1.33–3.26]; *p* = 0.001), and RVD (0.72 [0.58–0.89] per 1‐mm increase; *p* = 0.002). Indeed, cases with P3 segment involvement and a small RVD had a higher risk of restenosis regardless of cilostazol use (Figure 4). By Kaplan‐Meier analysis, freedom from restenosis by lesion risk factors is estimated in a population with (Figure 4A) and without cilostazol use (Figure 4B). Both Kaplan–Meier analyses indicate that any risk factors were associated with worse primary patency rate, compared with cases without risk factors. Combined use of a finalized device with another did not have any significant interaction effect between each other for the restenosis risk; the hazard ratios of the interaction term were 1.06 [0.45–2.47] between DCB and DES (*p* = 0.89), 0.60 [0.20–1.83] between DCB and SG (*p* = 0.37), 0.79 [0.22–2.82] between DCB vs. IWS (*p* = 0.71), 1.91 [0.80–4.61] between DES and SG (*p* = 0.15), 1.10 [0.43–2.83] between DES and IWS (*p* = 0.84), and 1.31 [0.41–4.20] between SG and IWS (*p* = 0.65), respectively. Table 3 summarizes the rate of periprocedural complications and 12‐month clinical outcomes. The all‐cause mortality, major amputation, and CD‐TLR rates were 9.7%, 1.8%, and 13.3% at 12 months, respectively.

**Table 2:** Association of baseline characteristics with restenosis risk

	Unadjusted hazard ratio	Adjusted hazard ratio
Male sex	0.74 [0.53−1.05] (*p* = 0.088)	N/I
Age (per 10‐year increase)	0.84 [0.70−1.01] (*p* = 0.060)	N/I
Diabetes mellitus	1.39 [0.97−1.99] (*p* = 0.070)	N/I
Smoking	0.84 [0.55−1.27] (*p* = 0.40)	N/I
Renal failure on dialysis	1.36 [0.94−1.95] (*p* = 0.10)	N/I
Dual antiplatelet therapy	0.64 [0.24−1.74] (*p* = 0.39)	N/I
Cilostazol use	0.47 [0.32−0.69] (*p* < 0.001)	0.49 [0.32−0.74] (*p* = 0.001)
Anticoagulant use	1.61 [1.06−2.45] (*p* = 0.024)	1.42 [0.92−2.20] (*p* = 0.12)
Chronic limb‐threatening ischemia	1.18 [0.83−1.68] (*p* = 0.36)	N/I
Ankle brachial index (per 0.1‐unit increase)	0.92 [0.87−0.98] (*p* = 0.012)	0.96 [0.90−1.02] (*p* = 0.21)
History of endovascular therapy	1.61 [0.99−2.62] (*p* = 0.054)	N/I
Popliteal involvement	0.98 [0.69−1.38] (*p* = 0.89)	N/I
P3 involvement	2.39 [1.63−3.50] (*p* < 0.001)	2.08 [1.33−3.26] (*p* = 0.001)
Reference vessel diameter (per 1‐mm increase)	0.62 [0.50−0.76] (*p* < 0.001)	0.72 [0.58−0.89] (*p* = 0.002)
Lesion length (per 100‐mm increase)	1.42 [1.05−1.92] (*p* = 0.022)	1.26 [0.89−1.77] (*p* = 0.19)
Chronic total occlusion	1.03 [0.72−1.49] (*p* = 0.86)	0.84 [0.67−1.06] (*p* = 0.15)
PACSS classification	0.96 [0.87−1.06] (*p* = 0.46)	N/I
Below‐the‐knee runoff	0.78 [0.63−0.96] (*p* = 0.022)	N/I
DCB use	1.37 [0.97−1.92] (*p* = 0.073)	N/I
DES use	0.89 [0.63−1.26] (*p* = 0.51)	N/I
Stent graft use	0.88 [0.60−1.29] (*p* = 0.52)	N/I
IWS use	1.43 [0.90−2.28] (*p* = 0.13)	N/I
Intravascular ultrasound use	0.57 [0.38−0.85] (*p* = 0.005)	0.70 [0.45−1.06] (*p* = 0.093)

*Note*: Data are hazard ratios [95% confidence intervals] (P values).

Abbreviations: DCB, drug‐coated balloon; DES, drug‐eluting stent; IWS, interwoven nitinol stent; N/I, not included; PACSS, Peripheral Arterial Calcium Scoring System.

**Table 3:** Perioperative complication and 12‐months clinical outcomes

Clinical outcomes	(*n* = 504)
Periprocedural complications	
Distal embolization	7 (1.4%)
Blood transfusion due to access site complications	2 (0.4%)
Arterial perforation	8 (1.6%)
Surgical conversion	1 (0.2%)
12‐month outcomes	
All‐cause mortality	49 (9.7%)
Major amputation	9 (1.8%)
Target lesion revascularization	67 (13.3%)
Surgical revascularization	1 (0.2%)

*Note*: Categorical variables are expressed as number and percentage.

## 4 DISCUSSION

This study evaluated the efficacy of EVT using the latest devices for patients with FP lesions longer than 25 cm. The main findings in the present study are as follows: (1) the 1‐year primary patency rate and freedom from CD‐TLR were 79.3% and 82.4%, respectively; (2) the predictors of 1‐year restenosis were the lack of cilostazol, P3 segment involvement, and small RVD; (3) all‐cause mortality, major amputation, and CD‐TLR were observed in 9.7%, 1.8%, and 13.3%, respectively, of the whole population at 1 year. The previous registries demonstrated that poor primary patency was associated with conservative antiplatelet therapies, involvement of popliteal segments and small vessel disease^7^, ^8^, ^17^, ^18^; however, these studies included patients with relatively short FP lesions (mean length ranged from 8.4 to 21.9 cm). Our study highlighted that the importance of the classical risk factors was consistent in extremely diffuse FP lesions. Furthermore, the current EVT devices provided acceptable primary patency rate in diffuse FP lesions.

The latest guideline recommends BSX using an autologous saphenous vein in patients with long (i.e., ≥25 cm) FP lesions as class I indication.^4^ Humbarger et al. demonstrated that FP‐BSX using a saphenous vein demonstrated satisfying clinical outcomes, demonstrating 85.3% occlusion‐free survival and 89.4% amputation‐free survival at 1 year follow‐up in 3930 patients.^19^ This study included operable patients with fewer comorbidities and a mean age of 65.5 years, among whom 48.0% had diabetes, 4.8% had chronic kidney disease, and 4.5% were on hemodialysis.^19^ Therefore, the guideline also recommended BSX in patients whose life expectancy could be more than 2 years.^4^ Because the recent patients with PAD tended to have multiple comorbidities due to advanced age,^5^, ^6^ patients with both high surgical risks and diffuse FP disease cannot always undergo BSX safely. The present study enrolled patients with diffuse FP lesions treated with EVT because of the high rates of advanced age (mean age was 76 years) and comorbidities such as diabetes (62.7%), and hemodialysis (27.4%). Although EVT is not recommended strongly for diffuse FP lesions, it may be notable that the 1‐year rates of all‐cause mortality and major amputation were quite low at 9.7% and 1.8% respectively in such population owing to the latest EVT devices and intensive medical treatment.

The previous real‐world registries demonstrated the feasible patency rate in diffuse FP lesions 1 year after EVT using the novel DES (87.1%),^17^ DCB (79.2%),^20^ SG (80.3%),^21^ and IWS (94.1%).^22^ Compared with these clinical trials using single types of devices, this study employed several current EVT devices at the discretion of each operator. Despite the potential bias in selecting the finalized devices in this study, the primary patency was 79.3% at 1 year, which was comparable to data from these registries.^17^, ^20^, ^21^, ^22^ Each finalized device does not have a similar performance, and it might be important to select the devices appropriately according to the baseline characteristics.^18^

In the present study, the primary patency rate was 90.3% at 1 year in cases on cilostazol treatment without small vessels and P3 segment involvement. On the contrary, numerous studies have shown that a small RVD and involvement of popliteal segments decreased the patency rate after EVT.^17^, ^18^, ^20^, ^21^ BSX is more suitable in diffuse FP lesions; however, popliteal involvement sometimes makes BSX difficult because a single‐segment great saphenous vein cannot cover the whole lesion and below‐knee anastomosis have to be performed.^23^, ^24^ Therefore, EVT has been conducted for such complex lesions in clinical practice. Small vessels can be related to evoking scaffold thrombosis formation, vessel recoil, and/or edge restenosis.^17^, ^18^ DES and SG have reliable antiproliferative barriers, which consisted of paclitaxel‐spacer technology, biocompatible fluoropolymer coating, or expanded polytetrafluoroethylene with a heparin bioactive surface to prevent vessel recoil and thrombus formation. To reduce edge restenosis, the previous study demonstrated the efficacy of additional DCB dilatation at scaffold edges.^25^ Popliteal segments are under great mechanical stress owing to its anatomical location, and has been considered a challenging vasculature because of the high rate of stent fracture.^26^, ^27^ IWS can adapt to arterial anatomies and may be one of the acceptable devices in popliteal segments.^28^ However, because IWS does not have an antiproliferative barrier like the abovementioned devices, the efficacy of IWS may be limited in diffuse FP lesions. Accordingly, when EVT is performed in diffuse FP lesions with small RVD and involvement of P3 segments, each EVT device should be selected according to lesion characteristics, and combination use of finalized devices may often be feasible for managing diffuse FP lesions.

This study suggested the efficacy of cilostazol administration for preventing restenosis in patients with diffuse FP lesions. The reduction of restenosis owing to cilostazol might be derived from its suppressive effect against neointimal proliferation, as well as from the vasodilator effects caused by inhibiting phosphodiesterase type 3, which results in increased blood flow in lower limbs.^7^, ^29^ A previous meta‐analysis indicated that cilostazol was related to a better primary patency after FP stenting.^29^ Furthermore, Zen et al. showed that cilostazol might contribute to a lower rate of 1‐year restenosis (95% CI 41%–62%, *p* = 0.008) in patients treated with implantation of drug‐coated stents.^7^ Diffuse lesions are still an unresolved issue in current FP‐EVT; therefore, additional cilostazol treatment might be helpful to reduce restenosis in patients with complex FP lesions.

## 5 STUDY LIMITATIONS

The present study has several limitations. First, this study was nonrandomized and retrospective. This study was also an exploratory analysis, and future pre‐specified studies would be needed to validate the present findings. Second, the EVT devices were selected according to discretion of each operator without pre‐established protocol, which might cause selection bias. Third, patient/lesion information, and angiographic findings were not evaluated by an external core laboratory. Forth, DAPT continued at least 1 month after EVT in this study; however, the accurate duration of antithrombotic treatment was unknown.

## 6 CONCLUSIONS

The current EVT might show the acceptable 1‐year primary patency rate at 79.3% in patients with FP lesions longer than 25 cm. A small vessel and P3 segment involvement might be associated with a poor patency rate 1 year after EVT. Cilostazol administration might contribute to reducing restenosis.

## AUTHOR CONTRIBUTIONS

**Kazunori Horie**: Conceptualization; data curation; investigation; methodology; project administration; writing – original draft. **Mitsuyoshi Takahara**: Formal analysis; supervision; visualization; writing – review and editing. **Tatsuya Nakama**: Conceptualization; data curation; investigation; supervision; writing – review and editing. **Akiko Tanaka**: Data curation; writing – review and editing. **Kazuki Tobita**: Data curation; writing – review and editing. **Naoki Hayakawa**: Data curation; writing – review and editing. **Shinsuke Mori**: Data curation; writing – review and editing. **Yo Iwata**: Data curation; Writing – review and editing. **Kenji Suzuki**: Data curation; investigation; methodology; project administration; supervision; writing – review and editing.

## CONFLICTS OF INTEREST

K. S. received remuneration for lecture from Boston Scientific Japan, and consultant fee from Medtronic. T. N. is a consultant of BD, Boston Scientific, Century Medical Inc., COOK Medical, Cordis Cardinal Health, Kaneka Medix, NIPRO, and OrbusNeich and received lecture fee from Abbot Vascular, Terumo and Medtronic. K. T. is a consultant of Gore and received Speakers' fee from Medtronic. The remaining authors declare no conflict of interest.

## ETHICS STATEMENT

This study was Approved from the local ethics committee. All procedures performed in studies involving human participants were in accordance with the ethical standards of the institutional and/or national research committee and with the 1964 Helsinki Declaration and its later amendments or comparable ethical standards.

## TRANSPARENCY STATEMENT

The lead author Kazunori Horie affirms that this manuscript is an honest, accurate, and transparent account of the study being reported; that no important aspects of the study have been omitted; and that any discrepancies from the study as planned (and, if relevant, registered) have been explained.

## Data Availability

The data that support the findings of this study are available on request from the corresponding author. The data are not publicly available due to privacy or ethical restrictions.
